# Production and Quality Control of [^67^Ga]-DOTA-trastuzumab for Radioimmunoscintigraphy

**Published:** 2013

**Authors:** Behrooz Alirezapour, Amir R. Jalilian, Fatemeh Bolourinovin, Sedigheh Moradkhani

**Affiliations:** a *Agricultural, Medical and Industrial Research School (AMIRS), Nuclear Science and Technology Research Institute, Rajaeeshahr, Karaj, Iran, P.O.Box: 31485-498.*; b *Nuclear Science Research School, Nuclear Science and Technology Research Institute, Tehran, 11365-3486, Iran. *

**Keywords:** Gallium-67, Trastuzumab, Radiolabeling, Conjugation, Biodistribution

## Abstract

Breast cancer radioimmunoscintigraphy targeting HER2/neu expression is a growing field of work in nuclear medicine research. In this study, trastuzumab was successively labeled with [^67^Ga] GaCl_3_ after conjugation with DOTA-NHS-ester.

The conjugates were purified by molecular filtration, the average number of DOTA conjugated per mAb was calculated and total concentration was determined by spectrophotometric method. DOTA-Trastuzumab was labeled with ^67^Ga. Radiochemical purity, integrity of protein after radiolabeling and stability of ^67^Ga-DOTA-Trastuzumab were determined followed by biodistribution studies in wild-type rats (30 ± 5.5 μCi, 2, 4 and 24 h p.i.).

The radioimmunoconjugate was prepared with a radiochemical purity of higher than 95% (RTLC). The average chelate to antibody ratio (c/a) for the conjugate used in this study was 5.8:1. The final compound was stable in presence of PBS at 37ºC and room temperature. The sample was showed to have similar patterns of migration in the gel electrophoresis similar to the native protein. The accumulation of the radiolabeled antibody in liver, spleen, kidney, heart and other tissues demonstrates.

^67^Ga-DOTA-Trastuzumab was prepared as a surrogate for important clinically applicable radionuclides used in SPECT and PET including In-111 and Cu-64 as a model of radiolabeling. It is also a potential compound for molecular imaging of SPECT for diagnosis and treatment studies and follow-up of HER2 expression in oncology.

## Introduction

The HER2/neu antigen is a transmembrane receptor (1) overexpressed in 25-30% of breast cancers (2). The overexpression has been implicated in the carcinogenesis of breast cancer and is an independent prognostic indicator of survival in patients (3). Trastuzumab is a humanized IgG1 monoclonal antibody (mAb) recognizing an epitope in the extracellular domain of the receptor and is used for immunotherapy for HER2/neu-positive tumors (4). 

Breast cancer radioimmunoscintigraphy targeting HER2/neu expression has been proposed by different research groups and could allow direct assessment of the receptor status of primary and metastatic lesions suggesting the effectiveness of Herceptin therapy. Herceptin and its fragments have been radiolabeled and used in the imaging of HER2/neu-positive tumors using In-111 (5) Y-90(6), Y-86 (7), Br-76 (8) and Zr-89 (9). The interesting physical properties and availability of gallium-67 make it an interesting nuclide for radiopharmaceutical research (10). Table 1 demonstrates the most important Ga radionuclide physical properties.

**Table 1:** Nuclear properties of Ga radionuclides

**Properties**	^67^ **Ga**	^68^ **Ga**	^66^ **Ga**
Gamma energy (keV)	3, 185, 300	511(*β*+)	511(*β*+), 834, 1039 2752
β-/β+ energy	84,92	1900(*β*+)	4153(*β*+)
Mode of decay	EC to ^67^Zn	10% EC to ^68^Zn, 90% *β*+	43% EC to ^66^Zn, 57% *β*+
Nuclear reaction	^68^Zn(p,2n)^67^Ga	^68^Ge Daughter ^66^Zn(*α*,2n)^68^Ge	^66^Zn(p,n)^66^Ga
Half-life	78 h	68 min	9.6 h
Natural abundance)%)	(18%)	(28%)	(28%)
possible contaminations	^66^Ga, ^65^Zn	^68^Ge	^65^Zn
Proton energy(MeV)	12-22	12-22	6-15

Due to the superior bioconjugation of DOTA bi-functional ligands N-succinimidyl-1,4,7,10-tetraazacyclododecane-1,4,7,10-tetraacetic acid (DOTA-NHS) was used as a bi-functional ligand (11). This ligand has already shown good biological performance when used in protein conjugation of various radioisotopes such as Ac-225 (12), Lu-177 (13) and lead radioisotopes (14).

We have recently reported some radiolabeled antiCD20 immunoconjugates for developing a successful labeling protocol (15, 16).

Although Ga-67 Trastuzumab is not a suitable radiolabeled monoclonal antibody for nuclear medicine applications, in order to develop the In-111 and Cu-64 radiolabeled Trastuzumab conjugates, the appropriate set-ups for the conjugation, labeling and quality assurance processes we intended to use an accessible radionuclide, *i.e. *Ga-67.

Ga-67 is a ferric cation mimic, resembling to In-111, Zr-89, Y-90 and most important of all therapeutic radionuclides including Sm-153, Lu-177 *etc.*

In order to develop Herceptin radioimmunoconjugates for using in imaging/therapeutic studies, DOTA-trastuzumab (Herceptin) was labeled by Ga-67 chloride for preliminary biodistribution studies in rats. The conjugate can be alternatively used in the production of therapeutic radionuclides after positive SPECT imaging using [^67^Ga]-DOTA-trastuzumab.

## Experimental

Enriched zinc-68 chloride with a purity of more than 95% was obtained from Ion Beam Separation Group at Agricultural, Medical and Industrial Research School (AMIRS). Production of ^67^Ga was performed at the Nuclear Medicine Research Group (AMIRS) 30 MeV cyclotron (Cyclone-30, IBA). NHS-DOTA was purchased from Macrocycles (NJ, USA). Trastuzumab (Herceptin) was a pharmaceutical sample purchased from Roche Co. Radio-chromatography was performed by using a Bioscan AR-2000 radio TLC scanner instrument (Bioscan, Paris, France). A high purity germanium (HPGe) detector coupled with a Canberra™ (model GC1020-7500SL) multichannel analyzer and a dose calibrator ISOMED 1010 (Dresden, Germany) were used for counting distributed activity in rat organs. Calculations were based on the 184 keV peak for ^67^Ga. All values were expressed as mean ± Standard Deviation (Mean ± SD) and the data were compared using student’s t-test. Statistical significance was defined as p < 0.05. Animal studies were performed in accordance with the United Kingdom Biological Council›s Guidelines on the Use of Living Animals in Scientific Investigations, 2^nd^ edn.

*Production of* ^67^ *Ga*

^68^Zn (p, 2n) ^67^Ga was used as the best nuclear reaction for the production of ^67^Ga. Other impurities could be removed in the radiochemical separation process. After the target bombardment process, chemical separation was carried out in no-carrier-added form. The irradiated target was dissolved in 10 M HCl (15 mL) and the solution was passed through a cation exchange resin (AG 50W, H+ form, mesh 200-400, h:10 cm, Ø:1.3 cm) which had been preconditioned by passing 25 mL of 9 M HCl. The column was then washed by 25 mL of 9M HCl at a rate of 1 mL/min to remove copper and zinc ions. To the eluent, 30 mL water plus about 100 mL of a 6 M HCl solution was added. The latter solution was loaded on another exchange resin (AG1X8 Cl- form, 100-200 mesh, h: 25 cm, Ø:1.7 cm) pretreated with 6 M HCl (100 mL). Finally, the gallium-67 was eluted as [^67^Ga] GaCl_3_ using 2 M HCl (50 mL); the whole process took about 60 min.

*Quality control of the product*

*Control of radionuclide purity*

Gamma spectroscopy of the final sample was carried out counting in an HPGe detector coupled to a Canberra^TM^ multi-channel analyzer for 1000 sec.

**Figure 1:**
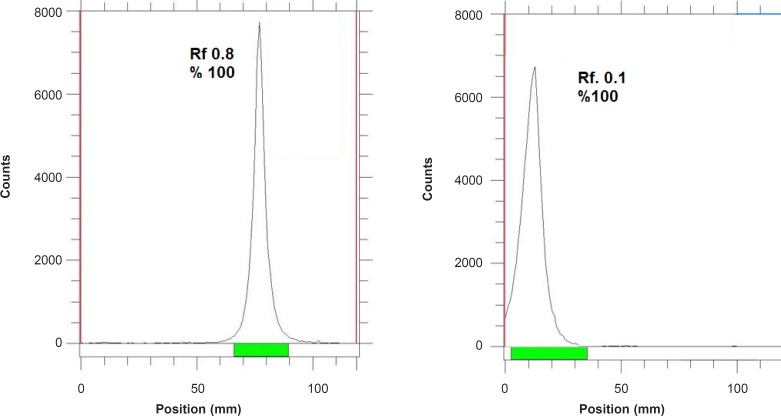
ITLC chromatograms of ^67^GaCl_3_ solution in DTPA solution (pH = 5) (left) and 10% ammonium acetate:methanol (1:1) solution (right) using Whatman NO. 2.

*Chemical purity control*

This step was carried out to ensure that the amounts of zinc and copper ions resulting from the target material and backing in the final product are acceptable regarding internationally accepted limits. Chemical purity was checked by differential-pulsed anodic stripping polarography. The detection limit of our system was 0.1 ppm for both zinc and copper ions.

*Conjugation of NHS-DOTA with the trastuzumab*

In the first step, lyophilized trastuzumab (Roche) was purified with water for injection from excipients by ultra-filtration. Vivaspin-2 filters (30 KDa; Sartorius AG; 2 × 10 min at 2.684 g) were used for all ultrafiltration purification steps. Briefly, trastuzumab was diluted with 0.2 M Na_2_CO_3_ (pH = 9.2) buffer solution. The antibody concentration was measured using a biophotometer (Eppendorf) at OD = 280 nm. The solution was passed through a Vivaspin 2 (20 min, 2.684 g) two times in order to remove the impurities. The antibody can then be removed from the upper part of the filter using bicarbonate buffer (0.2 M Na_2_CO_3_, pH = 9.2). The final concentration was re-measured using biophotometric assay as well as structure integrity test using SDS-PAGE. Then, DOTA-NHS (1.3 mg, excess 120 times) dissolved in bicarbonate buffer (400 μL, 0.2 M, pH = 9.2) was added to the purified antibody solution (3.3 mg/mL) in a borosilicate vial and mixed gently 20 times by pipetting. The mixture was gently shaken and incubated at room temperature for 24 h. The mixture was then transferred on a Vivaspin 2 cut-off filter (30KD) and centrifuged at 2.684 g for 15 min. In order to terminate the conjugation step and provide the suitable radiolabeling pH, the upper filter fraction is washed using ammonium acetate buffer (0.2M, pH = 5.5) three times in order to remove excess of DOTA-NHS. In this stage, to the upper fraction is added acetate buffer (1 mL) and the mixture is pipetted 10-20 times for immonoconjugate dissolution. The filter is then centrifuged upside-down at 2.684 g for 5 min. The antibody concentration was measured using a biophotometer (Eppendorf) at OD = 280 nm.

*Determination of the average chelate:antibody ratio*

The spectrophotometric method for quantitation of micromolar concentrations of bifunctional DOTA-NHS ligand in DOTA-monoclonal antibody (mAb) conjugates was performed according to the reported method (1). Briefly, the optical density of arsenazo yttrium (III) complex (2:1, 1 mL), prepared in 5.0 μM AAIII, 1.6 μM Y(III), 0.15 M sodium acetate buffer, pH 4.00, was measured at 652 nm. A standard curve was then plotted by the addition of multiple (8x) 15 μL DOTA-NHS standard solutions (DOTA-NHS dissolved in 0.15 M sodium acetate buffer, pH = 4.00), to the above mixture. In the second step, the optical density of 1:2 yttrium (III) complex of arsenazo (1 mL) was measured at 652 nm in the presence of conjugation product in order to determine DOTA-antibody attachments. 

*Radiolabeling of the antibody conjugate with* ^67^ *Ga*

Typically, 370 MBq of [^67^Ga] GaCl_3_ (in 0.2 M HCl) was added to a conical vial and dried under a flow of nitrogen. Acetate buffer (700 μL, pH = 5.5) was added to the gallium-containing vial and the vial was vortexed for 10 min. The conjugate containing fraction (500 μg) in acetate buffer with the measured protein content was added to the vial and mixed gently for 5 min using pipetting (10-20 x). The mixture is then incubated at 40ºC for 90 min followed by testing the radiochemical purity by ITLC using a radio TLC scanner (Whatman No.1, 1 mM DTPA). Finally, ETDA solution (10 μL, 10 mM) is added to the labeling mixture and incubated for 10 min in order to scavenge the unlabeled Ga cation. The mixture is then passed through the disposable PD10 De-salting column (Amersham) in order to further increase the radiochemical purity of the mixture. The final solution is then passed through a 0.22 micron biological filter for animal studies.

*SDS-polyacrylamide gel electrophoresis*

The radioimmunoconjugate was analyzed for integrity by SDS-polyacrylamide gel electrophoresis (SDS-PAGE). The radiolabeled mAb was evaluated with and without reduction by 2-mercaptoethanol. Approximately 200,000 cpm of each preparation was applied per lane and 4-20% polyacrylamide was run according to the method of Laemmli (2).

*Stability testing of the radiolabeled compound in final formulation*

Stability of [^67^Ga]-DOTA-trastuzumab in PBS was determined by storing the final solution at 4°C for 14 days (almost 5 times of physical half-life) and performing frequent ITLC analysis to determine radiochemical purity. The stability of the conjugated DOTA-trastuzumab stored at - 20°C for more than 1 month was also investigated. ITLC analysis of the conjugated product was performed to monitor the degradation products or other impurities. After subsequent ^67^Ga-labeling of the stored conjugated product, both labeling efficiency and radiochemical purity were determined. ITLC was performed by sampling the radiolabeled complex on a Whatman paper followed by developing in 1 mM DTPA aqueous solution.

*Measurement of transchelation to EDTA*

A 0.1 mL aliquot of the ^67^Ga-labeled product was mixed with 0.1 mL of 3 mM ethylenediaminetetraacetic acid (EDTA) (pH = 7.4) and allowed to be incubated for 30 min at room temperature. The entire solution was used for gel column scanning. For a control, another 0.1 mL aliquot was mixed with 0.1 mL of distilled water and also allowed to be incubated for 30 min at room temperature prior to gel column scanning. The protein labeling percentage was calculated using the following equation according to the reported method (3).

*Stability testing of the radiolabeled compound in the presence of human serum*

Radiolabel stability was assessed by size exclusion chromatography on a Sepharose column (1 × 30 cm). The column was equilibrated with PBS and eluted at a flow rate of 0.5 mL/min at room temperature; 1 mL of fractions was collected.

*Biodistribution of* ^67^ *Ga-DOTA-trastuzumab in wild-type rats*

To determine biodistribution, ^67^Ga-DOTA-trastuzumab and ^67^GaCl_3_ were administered to normal rats separately. A volume 50-100 μL of final radioactive solution containing 30 ± 5 μCi radioactivity was injected intravenously to rats through their tail vein. The total amount of radioactivity injected into each rat was measured by counting the 1 mL syringe before and after the injection in a dose calibrator with a fixed geometry. The animals were killed by CO_2_ asphyxiation (after anesthesia induction using propofol/xylazine mixture) at selected times after injection at the exact time intervals (6, 12 and 18 h for both samples) and the specific activities of different organs were calculated. Dissection began by drawing blood from the aorta, followed by collecting heart, spleen, kidneys, liver, intestine, stomach, lung, bone muscle and skin samples. The samples were weighed and their specific activities were determined with an HPGe detector counting the area under the curve of the 511 keV peak. The tissue uptakes were calculated as the percent of area under the curve of the related photo peak per gram of tissue (%ID/g). 

## Results and Discussion

*Radionuclide production*

Gallium-67, in form of GaCl_3_, was prepared by 24 MeV proton bombardment of the ^68^Zn target at Cyclone-30 on a regular basis. The target was bombarded with a current intensity of 170 μA and a charge of 1400 μAh. The chemical separation process was based on a no-carrier-added method. 

Radiochemical separation was performed by a two-step ion exchange chromatography method with a yield of higher than 95%. Quality control of the product was performed in two steps. Radionuclidic control showed the presence of 93(40%), 184(24%), 296(22%) and 378(7%) keV gamma energies, all originating from ^67^Ga and showed a radionuclidic purity higher than 99% (E.O.S.). The concentrations of zinc (from target material) and copper (from target support) were determined using polarography and shown to be below the internationally accepted levels*, i.e*. 0.1 ppm for Zn and Cu (4, 5). 

The radioisotope was dissolved in acidic media as a starting sample and was further diluted and evaporated for obtaining the desired pH and volume followed by sterile filtering. The radiochemical purity of the ^67^Ga solution was checked in two solvent systems. In 1 mM DTPA, free Ga^3+^ cation is converted to a more lipophilic GaDTPA form and migrates to higher R_f_ (0.8) while any small radioactive fraction remaining at the origin could be related to other Cu ionic species, not forming GaDTPA complex, such as GaCl_4_^-^, *etc*. and/or colloids (not observed). 

**Figure 2:**
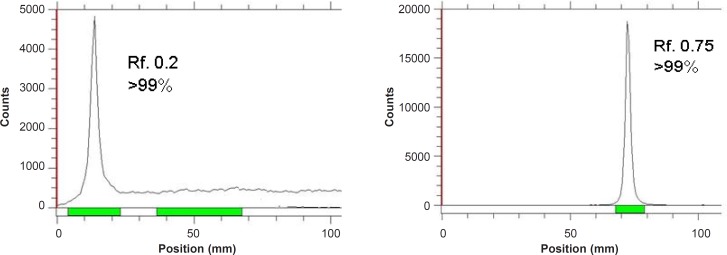
ITLC chromatograms of ^67^GaCl_3_ solution (right) and final ^67^Ga-DOTA-trastuzumab solution (left) on Whatman NO. 2 paper using 1 mM DTPA solution (pH = 5).

On the other hand, 10% ammonium acetate:methanol mixture was also used to determine radiochemical purity. The fast eluting species was possibly the ionic Ga-67 cations other than Ga^3+^ (not observed) and the remaining fraction at R_f_._0_ was a possible mixture of Ga^3+ ^and/or colloids. The difference in values of impurity in two solvent systems is possibly due to the presence of colloidal impurity in the sample (Figure 2), considering the purities of both chromatograms gallium cations are the only present radiochemical species. 

*Conjugation of trastuzumab with DOTA NHS and radiolabeling of trastuzumab with* ^67^ *Ga*

In order to overcome the effect of excipients and producing appropriate acidity for conjugation step, the pharmaceutical sample was purified by ultra-filtration using cut-off filters followed by determination of the antibody concentration using spectrophotometry. In order to improve the conjugation step, alkaline pH is necessary, thus, bicarbonate buffer was used to reconstitute the antibody. In each step, biophotometric assay as well as structure integrity test were performed in order to guarantee the quantity and the quality of the antibody, using SDS-PAGE. The use of polymer tubes and other synthetic materials in the conjugation and labeling step was interfered by the conjugation reaction, while borosilicate vials were the appropriate vessels. In order to remove the leftover of DOTA-NHS in the reaction and concentrate the antibody, the cut-off filter was used once more (30 KD).

At this stage, a buffer with pH of 5 was used to recover the antibody in order to terminate the conjugation step and provide the suitable radiolabeling pH, and for final fraction, the quantity of the antibody was measured at OD 280 nm.

In order to estimate the number of DOTA prosthetic group on each antibody molecule, the arsenazo yttrium complex (Y(AAIII)_2_) method was used. The absorbance of Y(AAIII)_2_ at 662 nm is decreased upon the addition of DOTA-trastuzumab while the corresponding absorbance of AAIII at 538 nm is increased. The Y(AAIII)_2_ and arsenazo III are the only absorbing species in solution; neither DOTA-trastuzumab nor its Y(III) complex have any absorbance in this wavelength region. The isosbestic point observed at 585 nm is consistent with only two absorbing species for reaction. The data demonstrated the DOTA:antibody ratio of 5.8:1.

The conjugated DOTA-trastuzumab fraction was mixed with ^67^GaCl_3_ solution at the appropriate acidity in acetate buffer at 40ºC for 90 min followed by testing the radiochemical purity by ITLC. By the addition of ETDA to the solution, the radiolabeling reaction as well as Ga-EDTA complex production were terminated, which can be better removed by size exclusion method. The EDTA scavenging time was shown to be critical in order to maintain the appropriate radiochemical purity. The increased EDTA incubation time led to decomposition of radioimmunoconjugate and the reduction of radiochemical purity.

*Radiochemical purity determination*

Instant thin layer chromatography using various mobile and stationary phases was performed in order to ensure the existence of only the desired radiolabeled antibody. Two different solvent systems with two stationary phases were tested. In all tests, radiolabeled antibody remained at the origin while other species migrated to other R_f_s depending on the mobile phase used. The R_f_s of the possible occurring chemical species in chromatography of the reaction steps are summarized in Table 1 (n = 5).

As shown in Table 2, for ^67^Ga^3+^ detection, the best eluent system is chromatographic protocol 2 resulting in Rf 0.9. For ^67^Ga-DOTA detection, system 1 can be used (R_f_. 0.3). ^67^Ga-DOTA-trastuzumab remains at the origin in all systems used due to the size and charge of the protein (≈150,000 D). Figure 2 demonstrates the ITLC chromatograms of ^67^GaCl_3_ solution and ^67^Ga-DOTA-trastuzumab solution. The mixture was further purified with desalting column to reach at least 90% radiochemical purity.

**Table 2:** The R_f _values of chemical impurities and DOTA-conjugates.

**Chromatography system**	**Chemical species**	**Mobile phase**	**Stationary phase**	R_f_
1	^67^Ga-DOTA-trastuzumab	10% Ammonium acetate:methanol (1:1)	Silicagel	0.0
	67Ga^2+^	//	//	0.0
	^67^Ga-DOTA	//	//	0.3
2	^67^Ga-DOTA-trastuzumab	1 mM DTPA (pH. 5)	Whatman No. 2	0.0
	^67^Ga^2+^	//	//	0.9
	^67^Ga-DOTA	//	//	0.0

*Stability of radiolabeled protein in presence of human serum in-vitro*

After incubation of [^67^Ga]-DOTA-trastuzumab (3.7 MBq) with freshly prepared human serum at 37°C for up to 72 h, 98% of all the radioactivity was eluted in the same position as [^67^Ga]-DOTA-trastuzumab, using size exclusion chromatography by means of G-50 Sephadex. Thus, there was no evidence for either degradation or transchelation of ^67^Ga to other serum proteins over a time period consistent with the normal blood clearance time of trastuzumab.

*Radiolabeled antibody challenge test*

The stability of [^67^Ga]-DOTA-trastuzumab (3.7 MBq) was demonstrated using challenge test (1). During the EDTA challenge test, two samples were diluted with 500-fold molar excess of disodium salt of EDTA solution in water, while two control samples were diluted with an equal amount of water. After 4 h of incubation at room temperature, all samples were analyzed using radio-ITLC.

*Protein integrity test using SDS-polyacrylamide gel electrophoresis*

**Figure 3:**
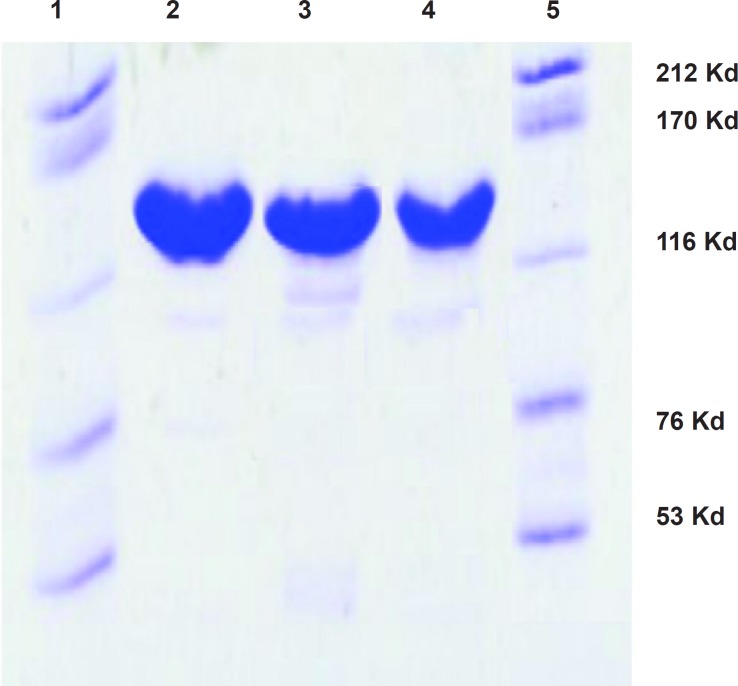
SDS-PAGE of the purified trastuzumab (Lane 2), conjugated DOTA-trastuzumab (Lane 3) and [^67^Ga]-DOTA-trastuzumab (Lane 4) monoclonal antibodies. Lanes 1 and 5, standard high molecular mass protein markers with molecular weights of 212, 170, 116, 76, and 53 KDa; all samples showed a molecular mass related to the whole IgG is 150 KDa.

^67^ *GaCl*_3_ *biodistribution in wild-type rat tissues*

In order to demonstrate the integrity of the protein after residulating and radiolabeling, gel electrophoresis was performed on the SDS PAGE gels using 16% bisacrylamide gel. The loaded samples were trastuzumab commercial sample, DOTA-trastuzumab and radiolabeled antibody samples four weeks after the experiment while kept in the fridge. Gels were stained with Coomassie Blue. The samples were showed to have similar pattern of migration in the gel electrophoresis reported previously (2) (Figure 3).

The liver uptake of the cation is comparable to many other radio-metals accumulation. About %10-15 of the activity is accumulated in the liver after 18 h. The transferrin-metal complex uptake and final liver delivery looks the possible route of accumulation.

**Figure 4:**
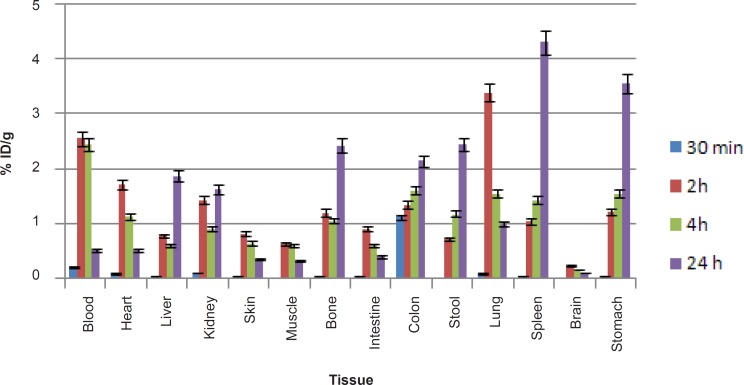
Biodistribution of [^67^Ga]GaCl_3_ (1.85 MBq, 50 μCi) in wild-type rats 0.5-24 h after iv injection via tail vein (ID/g%: percentage of injected dose per gram of tissue calculated based on the area under curve of 184 keV peak in gamma spectrum) (n = 5).

^67^ *Ga-DOTA-trastuzumab biodistribution in wild-type rat tissues*

For better comparison, biodistribution study was performed for free Ga^3+^. The %ID/g data are summarized in Figure 4. As reported previously, ^67^Ga is excreted majorly from gastrointestinal tract (GIT), thus colon and stool activity content are significant while blood stream activity is high at 2-4 h followed by reduction in 24. Bone uptake is also observed after 24 h post-injection.

**Figure 5:**
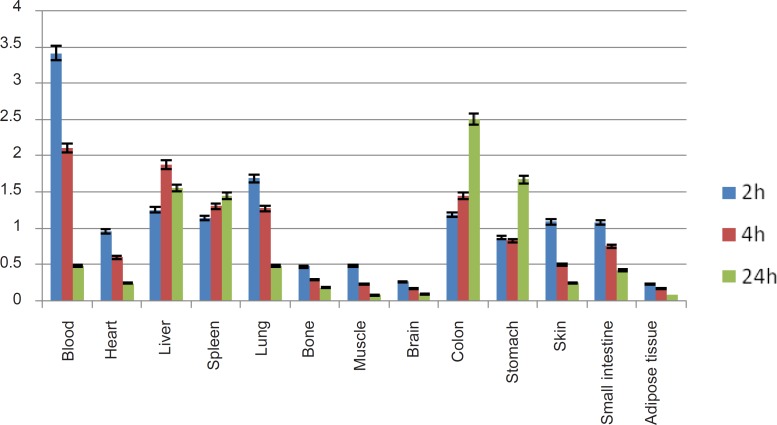
Bio-distribution of ^67^Ga-DOTA-trastuzumab in normal rats 2,4 and 24 h post-injection, kidney uptake is omitted.

As shown in Figure-5, high uptake in spleen and liver organs was observed that is due to the presence of HER2 antigens as well as protein accumulating property of the liver (%1.5-2) which in turn leads to high colon activity content. This has been already shown by other groups, working with ^125^I-anti-Her2 probes (1).

A significant blood content uptake is observed in Figure 5 which is also previously demonstrated by other groups working with ^67^Ga-labeled modified anti-Her2 probes using microPET imaging in nude mice (2). The latter work also reported high liver and heart accumulations 4 h p.i. which is consistent with our data. As shown in Figure 5, the heart is a medium accumulation site (about 1%).

The uptake data for ^67^Ga-DOTA-trastuzumab is comparable to ^89^Zr-trastuzumab (9), both ranging medium uptake for liver and in lower amount in intestine. However the bone uptake difference is a result of Zr-free cation affinity due to the metabolization of complex.

A detailed comparative study of both radioactive species demonstrates different organ biodistribution among the tissues during the study time. As shown in Figure 10, radiolabeled antibody is mainly accumulated in lungs after 18 h. On the other hand, gallium cation is slightly accumulated in the lungs starting from 6 to 12 h.

**Figure 6:**
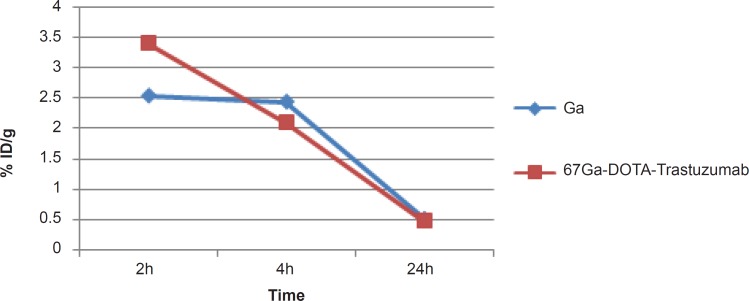
Comparative blood activity for ^67^GaCl_3_ and ^67^Ga-DOTA-trastuzumab in wild-type rats

Due to rapid gallium-scavenging property of transferrin as a homolog to ferric cation, in the serum and ultimate liver transfer of the gallium, the radiogallium cation is not observed in the circulation after 24 h (less than 0.5%) (Figure 6).

**Figure 7:**
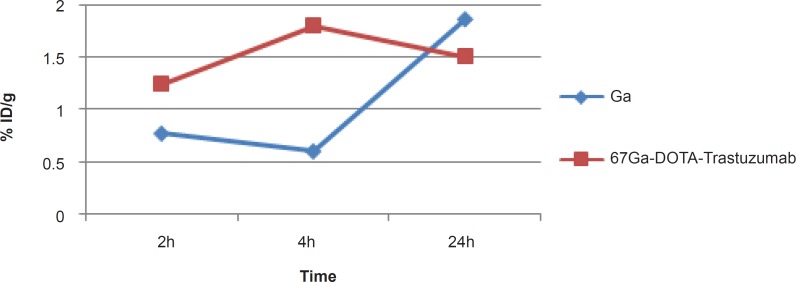
Comparative liver activity for ^67^GaCl_3_ and ^67^Ga-DOTA-trastuzumab in wild-type rats

In case of liver, radiolabeled antibody is mainly accumulated after 4 h, while it is almost constant in this organ after 24 h (1.5-2%). However in case of free gallium cation, it takes 24 h to reach around 2% close to that of radiolabeled antibody while the two species accumulate in liver via different mechanisms (Figure 7).

**Figure 8:**
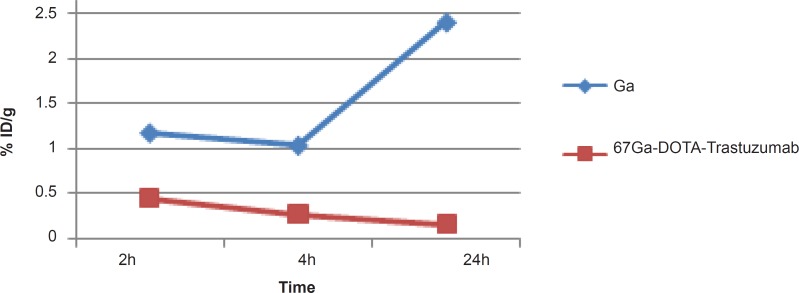
Comparative bone activity for ^67^GaCl_3_ and ^67^Ga-DOTA-Trastuzumab in wild-type rats

As shown in Figure 8, radiolabeled antibody does not accumulate in bone while gallium is majorly accumulated after 24 h due to the natural cation tendency to the anionic hydroxyapatite.

**Figure 9:**
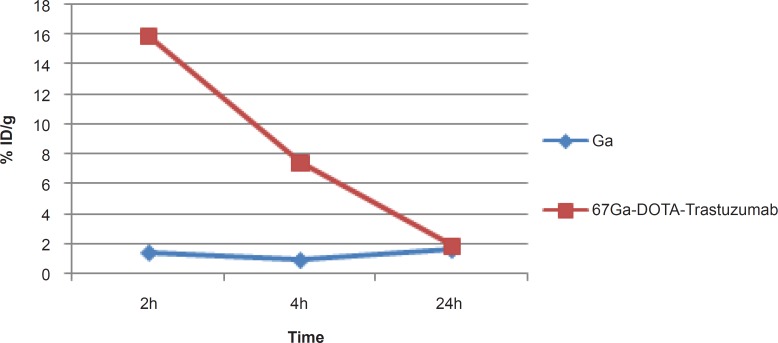
Comparative kidney activity for ^67^GaCl_3_ and ^67^Ga-DOTA-Trastuzumab in wild-type rats.

In case of kidney, radiolabeled antibody has significant uptake initially however, the activity is decreased after 24 h. Free gallium however is a natural liver accumulating cation leading to the gastrointestinal excretion of the activity, thus almost no activity is observed in the kidney at all of the time intervals. The significant kidney uptake in 2 h is possibly due to the presence of HER2 antigen. This was also previously demonstrated by other groups working with ^64^Cu-labeled modified anti-HER2 probes using microPET imaging in nude mice (23) (Figure 9).

## Conclusion

Radiochemical purity, integrity of protein after radiolabeling and stability of ^67^Ga-DOTA-Trastuzumab were determined followed by biodistribution studies in wild-type rats (30 ± 5.5 μCi, 2 h, 4 h, 24 h p.i.). The radioimmunoconjugate was prepared with a radiochemical purity of higher than 95% (RTLC) in 120 min. The average chelate to antibody ratio (c/a) for the conjugate used in this study was 5.8:1. The final compound was stable in presence of PBS at 37ºC and room temperature. The sample was showed to have similar patterns of migration in the gel electrophoresis. The conjugate retained the labeling efficacy after keeping at - /20oC after 1 month. The accumulation of the radiolabeled antibody in liver, spleen, kidney, heart and other tissues demonstrates a similar pattern to the other radiolabeled anti-HER2 immunoconjugates. ^67^Ga-DOTA-Trastuzumab is a potential compound for molecular imaging of SPECT for diagnosis and treatment studies and follow-up of HER2 expression in oncology. 

## References

[1] Schechter AL, Stern DF, Vaidyanathan L, Decker SJ, Drebin JA, Greene MI, Weinberg RA (1984). The neu oncogene: an erb-B-related gene encoding a 185,000-Mr tumour antigen. Nature.

[2] Pauletti G, Dandekar S, Rong H, Ramos L, Peng H, Seshadri R, Slamon DJ (2000). Assessment of methods for tissue-based detection of the HER-2/neu alteration in human breast cancer: a direct comparison of fluorescence in situ hybridization and immunohistochemistry. J. Clin. Oncol.

[3] Slamon DJ, Godolphin W, Jones LA, Holt JA, Wong SG, Keith DE, Levin WJ, Stuart SG, Udove J, Ullrich (1989). Studies of the HER-2/neu proto-oncogene in human breast and ovarian cancer. Science.

[4] Leonard DS, Hill ADK, Kelly L, Dijkstra B, McDermott E, O’Higgins NJ (2002). Anti-human epidermal growth factor receptor 2 monoclonal antibody therapy for breast cancer. Br. J. Surg.

[5] Tang Y, Wang J, Scollard DA, Mondal H, Holloway C, Kahnc HJ, Reilly RM (2005). Imaging of HER2/neu-positive BT-474 human breast cancer xenografts in athymic mice using 111In-trastuzumab (Herceptin) Fab fragments. Nucl. Med. Biol.

[6] Blend MJ, Stastny JJ, Swanson SM, Brechbiel MW (2004). Labeling anti- HER2/neu monoclonal antibodies with 111In and 90Y using abifunctional DTPA chelating agent. Cancer Biother Radiopharm.

[7] Garmestani K, Milenic DE, Plascjak PS, Brechbiel MW (2002). A new and convenient method for purification of 86Yusing a Sr(II) selective resin and comparison of biodistribution of 86Y and 111In labeled Herceptin. Nucl. Med. Biol.

[8] Winberg KJ, Persson M, Malmstrfm P-U, Sjfberg S, Tolmachev V (2004). Radiobromination of anti-HER2/neu/ErbB-2 monoclonal antibody using the p-isothiocyanatobenzene derivative of the [76Br]undecahydrobromo- 7,8-dicarba-nido-undecaborate(1-) ion. Nucl. Med. Biol.

[9] Dijkers ECF, Kosterink JGW, Rademaker AP, Perk LR, van Dongen GAMS, Bart J, de Jong JR, de Vries EGE, Lub-de Hooge MN (2009). Development and Characterization of Clinical-Grade 89Zr-Trastuzumab for HER2/neu ImmunoPET Imaging. J. Nucl. Med.

[10] Firestone RB, Shirley VS, Baglin CM, Zipkin J (1996). Table of isotopes.

[11] Chappell LL, Ma D, Milenic DE, Garmestani K, Venditto V, Beitzel MP, Brechbiel MW (2003). Synthesis and evaluation of novel bifunctional chelating agents based on 1,4,7,10-Tetraazacyclododecane-N,N,N,N-Tetraacetic acid for radiolabeling proteins. Nucl. Med. Biol.

[12] McDevitt MR, Ma D, Simon J, Frank K, Scheinberg DA (2002). Design and synthesis of 225Ac radioimmunopharmaceuticals. Applied Radiat Isotopes.

[13] Smith CJ, Galib H, Sieckmanc GL, Hayes DL, Owen NK, Mazuru DG, Volkert WA, Hoffman TJ (2003). Radiochemical investigations of 177Lu-DOTA-8-Aoc-BBN[7-14]NH2: an in- vitro/in-vivo assessment of the targeting ability of this new radiopharmaceutical for PC-3 human prostate cancer cells. Nucl. Med. Biol.

[14] Chappell LL, Dadachova E, Milenic DE, Garmestani K, Wu C, Brechbiel MW (2000). Synthesis, Characterization, and Evaluation of a Novel Bifunctional Chelating Agent for the Lead Isotopes 203Pb and 212Pb. Nucl. Med. Biol.

[15] Jalilian AR, Mirsadeghi L, Yari-kamrani Y, Rowshanfarzad P, Kamali-dehghan M, Sabet M (2007). Development of [64Cu]-DOTA-anti-CD20 for targeted therapy. J. Radioanal. Nucl. Chem.

[16] Yousefnia H, Radfar E, Jalilian AR, Bahrami-Samani A, Shirvani-Arani S, Arbabi A, Ghannadi-Maragheh M (2011). Development of 177Lu-DOTA-anti-CD20 for Radioimmunotherapy. J. Radioanal. Nucl. Chem.

[17] Fazaeli Y, Jalilian AR, Mohammadpour Amini M, Majdabadi A, Rahiminejad A, Bolourinovin F, Pouladi M (2012). Development of Ga-67 Maltolate Complex as an Imaging Agent. Iranian J. Pharm. Res.

[18] Jalilian AR, Yousefnia H, Shafaii K, Novinrouz A, Rajamand AA (2012). Preparation and Biodistribution Studies of a Radiogallium-AcetylacetonateBis (Thiosemicarbazone) Complex in Tumor-Bearing Rodents. Iranian J. Pharm. Res.

[19] Pippin CG, Parker TA, McMurry TJ, Brechbiel MW (1992). Spectrophotometric Method for the Determination of a Bifunctional DTPA Ligand in DTPA-Monoclonal Antibody Conjugates. Bioconlugate Chem.

[20] Laemmli UK (1970). Cleavage of structural proteins during assembly of the head of the bacteriophage T4. Nature (London).

[21] Rhodes BA, Zamora PO, Newell KD, Valdez EF (1986). Technetium-99m labeling of murine monoclonal antibody fragments. J. Nucl. Med.

[22] (2005). United States Pharmacopoeia 28.

[23] (2005). United States Pharmacopoeia 28.

[24] Tolmachev V, Velikyan I, Sandström M, Orlov A (2010). A HER2-binding Affibody molecule labelled with 68Ga for PET imaging: direct in-vivo comparison with the 111In-labelled analogue. Eur. J. Nucl. Med. Mol. Imaging.

[25] Spiridon CI, Sarah Guinn S, Vitetta ES (2004). A Comparison of the in-vitro and in-vivo Activities of IgG and F(ab′)2 Fragments of a Mixture of Three Monoclonal Anti-Her-2 Antibodies. Clin. Cancer Res.

[26] Dela Cruz JS, Trinh KR, Morrison SL, Penichet ML (2000). Recombinant Anti-Human HER2/neu IgG3-(GM-CSF) Fusion Protein Retains Antigen Specificity and Cytokine Function and Demonstrates Antitumor Activity. J. Immunol.

[27] http://www.wmicmeeting.org/2010/Abstracts/forSystemUse/papers/0107.html.

